# Promotion of environmental public health and environmental justice in communities affected by large and long lasting industrial contamination: methods applied and lessons learned from the case study of Porto Torres (Italy)

**DOI:** 10.3389/fpubh.2024.1408127

**Published:** 2024-07-10

**Authors:** Roberto Pasetto, Amerigo Zona, Daniela Marsili, Franca M. Buratti, Ivano Iavarone, Maria Eleonora Soggiu, Emanuela Testai

**Affiliations:** ^1^Environmental and Social Epidemiology Unit, Department of Environment and Health, Istituto Superiore di Sanità, Rome, Italy; ^2^WHO Collaborating Centre for Environmental Health in Contaminated Sites, Rome, Italy; ^3^Mechanisms, Biomarkers and Models Unit, Department of Environment and Health, Istituto Superiore di Sanità, Rome, Italy; ^4^Exposure to Air, Soil Contaminants and Lifestyle Unit, Department of Environment and Health, Istituto Superiore di Sanità, Rome, Italy

**Keywords:** public health, industrial pollution, environmental health, environmental justice, environmental exposure, toxicology, environmental epidemiology, environmental public health communication

## Abstract

**Introduction:**

Communities affected by large scale and long lasting industrial contamination are often keen to understand whether their health has been impaired by such contamination. This requires answers that integrate environmental public health and environmental justice perspectives. At these sites, exposure scenarios from environmental contamination over time by multiple chemicals, often involving different environmental matrices, are complex and challenging to reconstruct.

**Methods:**

An approach for describing the health of such communities in association with environmental contamination is presented, with the methods applied across the three domains of environmental contamination, population exposure and toxicology, environmental and social epidemiology, and environmental public health communication. The approach is described with examples from its application to the case study of Porto Torres, a town with a substantial industrially conditioned evolution.

**Results:**

Activities in the field of environmental contamination, population exposure and toxicology focus on the collection and systematization of available contamination data, the identification of priority pollutants based on their toxicological profiles, the qualitative assessment of the likelihood of exposure for the population to priority pollutants and their known health effects. Environmental and social epidemiology methods are applied to describe the health profiles and socioeconomic conditions of the local population, taking into account multiple health outcomes from local information systems and considering specific diseases based on exposure and toxicological assessments. The environmental public health communication methods are directed to produce a communication plan and for its implementation through interaction with local institutional and social actors. The interpretation of health profiles benefits from a transdisciplinary analysis of the results.

**Discussion:**

The proposed approach combines the needs of environmental public health and environmental justice allowing the integration of multidisciplinary knowledge to define recommendations for reducing and/or preventing hazardous environmental exposures and adverse health effects, stimulating the interactions between stakeholders, and making the study results more accessible to citizens.

## 1 Introduction

The topic of contaminated sites was included for the first time among the priorities in environmental health for the 53 countries of the WHO European Region in 2017 (1) and confirmed as a priority for action at the WHO European Region Ministerial Conference on environment and health in 2023 (2).

The large network of experts from 33 of the European COST Action “Industrially contaminated sites and health network” active in the period 2025–2019 defined contaminated sites as “areas hosting or having hosted industrial human activities which have produced or might produce, directly or indirectly (waste disposals), chemical contamination of soil, surface or ground-water, air, food-chain, resulting or being able to result in human health impacts” (3). This health-centered definition builds on an operational description of contaminated sites proposed by WHO (4), and may not coincide with other ones based on environmental damage/impact; for example, the European Environment Agency's inventory for contaminated sites refers to soil and water contamination only (5).

Contaminated sites vary greatly in both size and type of contamination. They range from areas affected by contamination with a single chemical in one environmental matrix (e.g., soil contamination caused by a specific pesticide) to large areas contaminated by multiple chemicals affecting soil, water, air, and the food chain (e.g., contamination caused by the long-term activities of a petrochemical complex).

In contaminated sites, pollution from various environmental sources can coexist and be closely interrelated with social determinants of the health of local communities. This is particularly evident in towns where the presence of large industries or industrial districts has led to a substantial industrially conditioned evolution, preventing them from pursuing other forms of development.

Communities living close to heavily contaminated industrial areas often experience socioeconomic deprivation and exhibit health profiles that show various excess risks and therefore can be considered overburdened communities (6). In Italy this phenomenon has been documented by the national epidemiological surveillance system of communities residing near the main national contaminated sites of interest for remediation, named SENTIERI (National Epidemiological Study of Territories and Settlements Exposed to Pollution Risk), implemented by the Italian National Institute of Health (ISS) (7, 8). SENTIERI has also highlighted a clear North-South gradient divide, with the worst conditions in the South Italy and big Islands (i.e., the Sicily and Sardinia Regions), where most of the affected communities also experience socioeconomic deprivation (8, 9). This gradient goes hand in hand with similar gradients of disadvantage observed at the national level that have complex historical reasons preceding and/or concomitant with the Italian industrialization process (10), as documented, for example, in the reports of the Italian National Institute of Statistics (ISTAT) on equitable and sustainable wellbeing (11).

SENTIERI is a system based on informative flows of health data, managed at the national level. It does not rely upon elaboration of locally collected environmental and health information, nor includes direct interaction with local stakeholders to build up shared study protocols and to account for perception and expectations of directly interested communities. Therefore, in order to better characterize analyses of local contexts the surveillance approach should be complemented with participatory studies with detailed assessments designed to catch the specificity of each contaminated site and including a better definition of the relevant exposures to environment and social risk factors in the potentially impacted population.

Promoting environmental public health in communities living near polluted industrial sites can directly contribute to improving environmental justice at the local level. Environmental justice has been defined in a variety of ways (12, 13), but in practice, it refers to the equitable allocation of environmental risks and benefits. In contaminated sites, the potential for environmental injustice arises when local communities and population groups are disproportionately affected within their respective administrative jurisdictions (e.g., Region/Province). This discrepancy is often associated with disadvantaged conditions, such as socioeconomic deprivation, and may be exacerbated by the social composition of these communities, including a high proportion of a particular ethnicity. As a result, these communities face increased environmental risks and fewer environmental benefits.

Environmental injustice, associated with industrially contaminated sites, has been frequently observed in assessed cases. This phenomenon started in the United States, where the concept of Environmental Justice originated (14). It has been studied through analyses on single sites (such as those documented by the journal “Environmental Justice”) (15) and through assessments at the national level (16). In the Italian context, environmental justice concerns about contaminated sites have been recently formalized by scholars, with some communities affected by contaminated sites recognized as “environmental justice communities” (17).

This paper aims to propose an approach able to characterize the health profiles of communities living close to industrial contaminated sites by integrating environmental, toxicological, epidemiological, and communication methodologies, and by interacting with local institutional and social actors. The integration of environmental, social and health disciplines with public health communication refers to the environment and health paradigm where “human health depends on the quality of our environment” (18). This paradigm underscores a continuous sequence of events linking pollution sources, environmental exposures and resulting health effects, where chemically contaminated areas and resident communities serve as examples of a real-life worst-case scenarios.

The design of an *ad hoc* integrated approach to deal with the complex environmental health scenarios posed by contaminated sites is critical, as they are often characterized by contamination processes and presumably exposure via multiple pathways to the local population persists for decades. Furthermore, robust quantitative data for reconstructing long-term exposure in the local population is often lacking (3). In some cases, no previous studies have characterized the health risks of the population in association with such environmental pressures, despite the local community requesting studies to clarify potential associations between contamination and their health profiles.

The local populations considered are composed of tens of thousands of people, and their evolution over time has been largely determined by the nearby industrial areas. Such a demographic dimension usually corresponds to “real” communities where people have the following characteristics: (i) a common geographical location; (ii) common interests and identities; (iii) social interactions that bind people together through reciprocal relationships; (iv) common needs and problems that can be addressed through collective action (19). The presence of these elements does not mean that communities are homogeneous and simple entities. The degree of cohesion depends on the ability of the community to respond to the needs of its members, as well as on its structural characteristics (20). In such contexts, public health researchers and practitioners can be encouraged to expand their interest in community-based approaches to public health promotion and disease prevention, as the presence of locally specific environmental and socioeconomic factors that are common to the community are combined with general factors of interest for community-level interventions, such as the complex etiology of health problems, the interplay between humans and their environment, and the limits to fostering individually-oriented strategies for behavior change (21).

The approach herein proposed is presented with its methodological framework and applications taking as a highly representative case-study the contaminated site of Porto Torres (Sardinia, Italy). Lessons learned from its application are reviewed under the dual perspectives of environmental public health and environmental justice.

### 1.1 Description of the historical evolution of the contaminated area of Porto Torres

Prior to describing the detailed features of the application of the proposed approach, it is beneficial to provide a brief overview of the site and its characteristics and historical background.

Porto Torres is a municipality of about 22,000 residents, located in the northwest of the Sardinia Region in Italy. The town of Porto Torres is in immediate proximity to a large industrial complex, which formerly included several petrochemical plants, a thermoelectric power plant, and an industrial and civil harbor.

Due to its historical development, Porto Torres can be considered a town that has undergone a substantial industrially conditioned evolution. The petrochemical industry began operating in the 1960s, reaching a peak of ~8,000 workers in the 1970s. The population of Porto Torres grew by ~45% in the first decade after the start of industrial activities.

Industrialization caused a deep transformation at the local level. In a short time, Porto Torres became one of the most important hubs of the European petrochemical industry, leading to the establishment of numerous associated companies operating both in production and in services.

Industrialization in the area led to significant changes in the local socio-economic context, including significant migration of inhabitants from inland areas toward the industrialized area. In the first decade of activities, employment in the industrial sector grew, accompanied by a corresponding decrease in the agricultural sector; the level of education increased, as evidenced by the decrease in the proportion of illiterate individuals in the years of full activity of the petrochemical site (1961–1981) (22).

Similarly, to other sites in Southern Italy and the major islands, relevant environmental contamination progressively became documented after the first period of industrial and socioeconomic development (8).

In 2002, Porto Torres was declared a site of national priority for remediation by the Ministry of Environment. Most of the petrochemical plants closed around 2010, resulting in the loss of much of the workforce, and the surviving activities currently involve a few 100 workers. According to the 2011 Census data, 80% of the local population resides in highly socioeconomically deprived census tracts (23).

## 2 Materials and methods

The approach applied in Porto Torres is based on the contribution of methods from the three domains of “environmental contamination, population exposure and toxicology,” “environmental and social epidemiology” and “environmental public health communication.” The domain concerning “environmental contamination, population exposure and toxicology” focuses on the collection and systematization of environmental data, the identification of priority pollutants based on their toxicological profiles, the qualitative attribution of the likelihood of exposure for the population and their known health effects. The “environmental and social epidemiology” domain refers to the methods applied to describe the health profiles and socioeconomic conditions of the local community, considering multiple health outcomes from local information systems. The “environmental public health communication” domain addresses the design and implementation of a contextualized participative communication plan.

**Figure 1:**
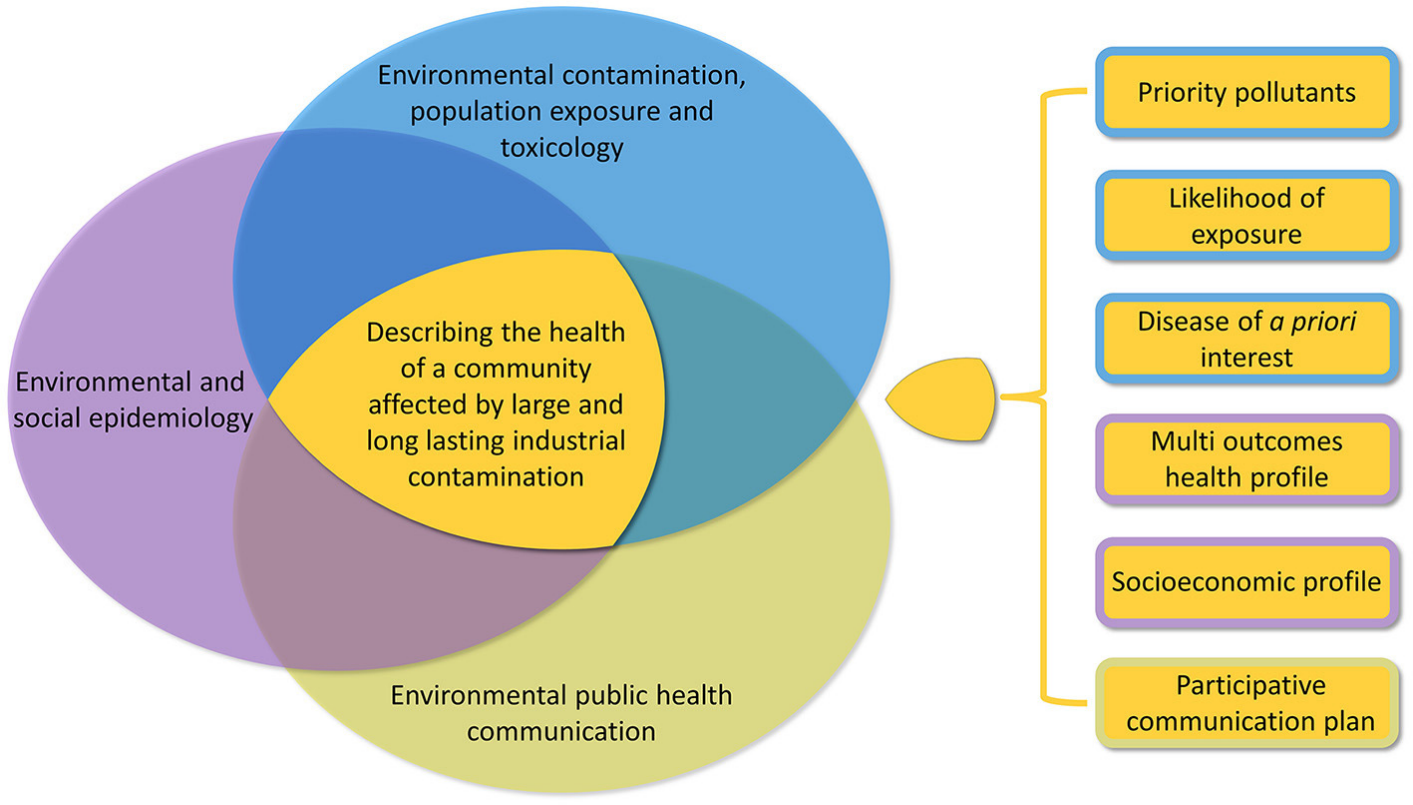
Approach of the study integrating the three research areas of “environmental contamination, population exposure and toxicology,” “environmental and social epidemiology,” and “environmental public health communication” and their outcomes.

Figure 1 presents an overview of the study approach, which represents the current update to the methodology originally developed and applied when the SENTIERI monitoring system was first activated (24). At that time, around 2010, the approach included only the field of environmental epidemiology, with the identification of diseases of *a priori* interest based on the review of epidemiological evidence on the risk for the population living near different sources of contamination (24). Such diseases were screened at area level using routinely collected mortality and morbidity data. A similar approach was used to analyse individual populations affected by contaminated sites, as a new descriptive approach complementary to analytical epidemiological studies to analyse the associations between a given source of contamination and the health of the affected population (25). Subsequent updates introduced the selection of priority pollutants for each site to define the diseases of *a priori* interest, depending not only on the sources of contamination, but also taking into account the local evidence on contamination (26) and the consideration of communication aspects (27). The importance of combining descriptive approaches with analytical ones to assess health risk and impact of contaminations was stressed within the European COST Action “Industrially contaminated sites and health network” (3, 28).

In the following sections the methods regarding the three domains as applied in the Porto Torres case study are described.

### 2.1 Environmental contamination, population exposure and toxicology

The collection and organization of environmental contamination data are of pivotal importance to have a comprehensive understanding of environmental degradation, as well as for exposure of the resident population.

In the Porto Torres area, the collection of data was conducted by asking to the regional environmental and health authorities to provide all the available documentation on environmental contamination within the municipality, including routine monitoring data, information from specific contamination events like fires or accidents, and reports from companies mandated to conduct monitoring activities within the industrial areas. The information were compiled, prioritizing georeferenced data. All gathered information was evaluated and a table was compiled, listing compounds exceeding Contamination Threshold Concentrations (CSCs) for soil and water. These concentrations, which are legally binding, serve as the threshold values triggering site characterization and risk assessment by the competent authority in Italy (i.e., the Ministry of Environment), in consultation with the Italian National Institute of Health regarding potential human health risks. This approach aligns with the Italian legislation on remediation of contaminated sites, which requires the chemical characterization of soil, groundwater, surface water bodies and their sediments, with the identification of contaminants whose concentrations exceed the threshold values expressed as CSC. As for the industrial emissions to the atmosphere, these are relevant not only for direct inhalation exposure (29, 30), but also for the possible concomitant contamination of soil and water bodies through fallout and deposition. This can potentially lead to the resident population being subject to aggregate exposure of a single contaminant via different routes and/or cumulative exposure to several contaminants. Considering the site's industrial history, current activities, chemical characterization for remediation purposes, potential exposure scenarios, likelihood of exposure and hazard characterization (in terms of inducing benign and/or malignant pathologies, taking into account exposure route-related effects, and any available information on dose-response effects) has led to selecting from that list a sub-list of chemicals identified as the “priority chemicals” for the Porto Torres population substantially following the methodology applied in SENTIERI (7, 26), considering a number of criteria, among which:

- the quality of the data and its origin,
- the presence of active emission sources of that substance,
- the co-occurrence of the pollutant in several environmental matrices (multi-exposure),
- the extent to which the environmental threshold (CSC) value is exceeded in the matrices,
- the substance's bioaccumulation and persistence properties,
- the hazard for human health (based on classification descriptors).

For the selected substances, pathologies of interest were identified based on biological plausibility, coming from the hazard characterization on available scientific evidence. The objective of this assessment was extended beyond carcinogenicity, to include non-carcinogenic toxic effects as well. In both cases, information on the target organs as well as the critical exposure routes (oral, dermal and inhalation), is essential, as different routes may lead to varying health effects. In the first step, the focus was on identifying the hazard of each substance (which is reflected in its classification): this approach is relevant for identifying all the potential targets. However, to go a step forward, the dose-response relationship and the derived “health based reference values” (HBV) were considered, to be compared with the level of exposure and assess the risk. Indeed, while the hazard represents an intrinsic characteristic of a substance, risk indicates the probability of experiencing a toxic event at a given exposure level. Thus, while a substance may be classified as highly toxic based on its hazard, if there's minimal probability for exposure over a lifetime, or if the substance has low absorption potential upon exposure, the risk to the population is likely negligible. For non-genotoxic substances, including carcinogens with known non-genotoxic mechanisms of carcinogenicity, a threshold dose is assumed (defining the above-mentioned HBV). This implies that exposure levels below this threshold do not result in adverse effects (31). Therefore, understanding the level of exposure and the dose-response relationship is crucial for quantitatively assessing health risks and correctly interpreting epidemiological findings.

The information regarding the toxicological profile has been gathered from internationally accredited sources, such as the International Agency for Research on Cancer (IARC), WHO, US National Toxicology Program (NTP), US Environmental Protection Agency (USEPA), and the European Union (EU) Commission and its relevant bodies (e.g., EFSA and ECHA). Each of these agencies and organization work according to established internationally agreed procedures for hazard and risk assessment (31). However, they often use their own classification schemes and specific descriptors, particularly regarding carcinogenicity. This is the case of carcinogen classification by IARC, the main reference for the assessment of potential carcinogenicity exclusively based on hazard. In addition to specific monographs (https://monographs.iarc.who.int/), IARC periodically updates tables with cancer sites and related agents for which a causal link has been recognized. IARC also identifies, whenever possible, the underlined mechanism by analyzing the genotoxic characteristics: this is important since for genotoxic carcinogens it is conventionally assumed that a threshold cannot be identified, and the risk assessment follows different methodologies (31). For some pathologies, the latency time between exposure and adverse outcomes are particularly important, consequently information on contamination dated back in time may be relevant.

Exposure assessment is a site-specific procedure that starts from the characterization of the human activities present in the area, the chemical-physical characteristics of the substances used/emitted and their emission scenario. The characteristics of the area (e.g., orography, meteorology, characterization and spatial distribution of the population), the environmental fate of pollutants and their occurrence in the different matrices as well as the habits of the population describe the exposure scenarios, allowing to estimate the population exposure for each exposure routes (32). To estimate the population's exposure dose, the appropriate exposure scenarios are to be built up to quantify inhalation and/or oral and/or dermal exposure. The potential exposure to the substance by different routes (aggregate exposure), as well as the assessment of exposure to several substances (or mixtures) for one or more routes (cumulative exposure) is another level of complexity in exposure assessment (33, 34).

### 2.2 Environmental and social epidemiology

#### 2.2.1 Health profiles

The approach used for providing health profiles derives from the methodology used in the national epidemiological surveillance program SENTIERI (7, 24). In the case of Porto Torres, health data were collected exclusively on the basis on the local health information systems and updated as much as possible over the time span of the study. Selected health outcomes include mortality, hospitalization, and cancer incidence.

For Porto Torres, health profiles at the community level derives from routinely collected on hospital discharges and data on mortality and cancer incidence from registries. Such data were collected based on local health information systems: mortality data from the Local Health Authority's registry of causes of death, hospitalization data from the Sardinian regional database on Hospital Discharge records, and cancer incidence data from the cancer registry of the province of Sassari. The health profile is described both in general and specific terms (7, 24, 26). The former includes all diseases, all malignant tumors, digestive diseases, cardiovascular diseases, respiratory diseases, genitourinary diseases, and diseases of the central nervous system. The latter focuses on diseases that epidemiological literature associates with residence close to contamination sources (in the case of Porto Torres, “petrochemical plants and refinery”) (see Table 1), and diseases identified through the described procedure for selecting contaminants present in the area. The purpose of describing the specific health profiles is to assess the health status of the local populations with reference to diseases of *a priori* interest and therefore to better contribute to verifying potential associations with local contaminations. Inizio modulo THE specific health profile is therefore a key step especially if it highlights excesses of risk regarding multiple pathologies/health outcomes in both sexes suggesting a possibly relevant role of environmental exposures. Results were stratified by sex and age (including developmental ages and young adulthood (< 1 year; 0–14 years, 15–29 years, and 0–29 years). Indirect Standardized Ratios, commonly used to describe health profiles, were calculated by identifying residents of the local province (Sassari) excluding the municipality of Porto Torres as the reference population. The Indirect Standardized Ratio for each health outcome and disease represents the ratio between observed cases in the target population (Porto Torres) and expected cases estimated in that population by applying age-specific provincial rates in the study period. For standardization, 5-year age classes are considered, excluding the first (< 1 year) and last (over 95 years) age classes. Confidence intervals at 90% are associated with each estimate and calculated using the Poisson formula for a number of observed cases < 100, and Byar's approximation for a number of observed cases equal to or higher than 100. In the case of Porto Torres, indicators to describe health profiles were calculated for a period of 10 years for each outcome.

**Table 1:** Diseases of *a priori* interest on the basis of epidemiological evidence from meta-analysis for risk associated with residence in proximity to petrochemical plants or for occupation in petrochemical plants (35–40) [+ excess in risk; (+) excess in risk with no statistical significance] [modified from Pasetto et al. (23)].

**Cause**	**Mortality**	**Prevalence**	**Cancer incidence**	**Evidence in SENTIERI 2010^§^**
**Residential exposure**				
All malignant cancers				Limited
Lung cancer	(+)		+	Limited
Leukemia			+	
Respiratory simpthoms		+		Limited for all diseases of the respiratory system combined, and acute respiratory diseases
Asthma		(+)		Limited
Childhood leukemia			(+)^*^	
Brain cancer			(+)^*^	
**Occupational exposure**				
Mesothelioma	+		+	
Cutaneous melanoma	(+)		+	
Gallbladder tumor	+			
Bladder cancer			+	
Kidney cancer	(+)		(+)	
Brain cancer	(+)			
Multiple myeloma	(+)		+	

Epidemiological evidence assessed within SENTIERI in association with petrochemical plants and refineries.

^§^The evidence from SENTIERI was updated in 2023 (7) including other outcomes classified as with Limited evidence: leukemia, cancer of connective tissue and other soft tissues, breast cancer.

^*^Estimates from a meta-analysis based on two studies only.

Temporal trends were analyzed for overall mortality and mortality for all tumors by sex. This was achieved by modeling standardized mortality rates and comparing figures for Porto Torres with those of the provincial reference population, following an approach based on graphical comparison of the Local Estimated Scatterplot Smoothing (LOESS) curves built on the time series of the rates described by La Serra et al. (41).

#### 2.2.2 Socioeconomic conditions

In the case of Porto Torres, social and socioeconomic conditions were assessed both qualitatively and quantitatively. Qualitative assessment involved analyzing documents and using historical data from censuses regarding socioeconomic variables in the municipality of Porto Torres (23) and through semi-structured interviews with some members of the city council (see Section 3.4). Quantitative assessment was based on two indicators: (1) the proportion of the population residing in highly deprived neighborhoods, using a deprivation index based on census data at the census tract level (42); (2) the standardized mortality ratio for premature mortality including main chronic non-communicable diseases potentially preventable, at least in part, in the age class 30–69 years (i.e., cardiovascular diseases, tumors, diabetes, and chronic respiratory diseases). This indicator was chosen to decline at the local level what is proposed in the framework of the UN Sustainable Development Goals to assess countries' progress toward each goal [UN indicator 3.4.1 - (43)]. Premature mortality helps to assess the vulnerability of a population, which is influenced by the effectiveness of health prevention and promotion policies and the quality of health care. It is also associated with socio-economic deprivation at both individual and contextual levels.

### 2.3 Environmental public health communication

The design of a participative communication plan associated with the epidemiological study was based on the methodological approach developed in SENTIERI (27, 44). This plan is implemented in four strategic steps taking into account the local social context: (i) identification of the communication aims considering the existing local sociocultural framework, (ii) identification and engagement of the local institutional and social actors in the activities aimed at communicating the study findings, (iii) selection of communication channels and the elaboration of tailored communication materials for diverse stakeholders, (iv) assessment of the impact of communication activities.

Considering the sociocultural context of the affected community when defining the goals of the communication plan is of paramount importance, as it influences individual and collective behavior and critical thinking (45). In the case of the Porto Torres community, two main objectives were identified. The first one aimed to disseminate and share new scientific information and knowledge with the entire community to improve its environmental health literacy; this involves empowering the community's capacity to be aware and use available information to proactively participate in the communication process (27, 46). The second objective, closely related to the first, was to promote an intersectoral network of local institutional actors to be involved in the participatory communication process on environmental health issues.

### 2.4 Integration of the domains

In the proposed approach, the integration of exposure assessment with toxicological and epidemiological information and data does not follow from data modeling. Rather, it is achieved through a deductive approach that examines the interrelationships between events, considering concurrence (e.g., temporal association between exposure and health effects), biological plausibility, and, whenever possible, other evidence supporting causality. It starts from the assessment of the characteristics of the contaminated site in terms of sources of pollution, and evidence on contamination, then it provides an evaluation on the likelihood of exposure to pollutants (via different exposure routes), using toxicology to identify potential health effects to be expected, and it ends focusing on the epidemiological description of health profiles at community level for diseases of *a priori* interest, selected on the basis of previous evidence in the other domains. Finally, the results on the health profiles are commented, combining exposure assessment, toxicological, and epidemiological reasoning.

In the Section 3, the methods applied in each area are presented in their theoretical rationale, followed by a description of the specific application in the case study with some examples. Finally, a selection of results from the Porto Torres application were used to show: (1) how to carry out an integrated evaluation of health profiles combining the contribution of environmental, toxicological, and epidemiological reasoning; (2) the limitations and potentialities in discussing the temporal trends of health profiles; (3) how the socioeconomic assessment should be considered under the environmental justice lens; (4) the main key features aspects of environmental public health communication with the community.

## 3 Results

### 3.1 Analysis of environmental contamination data

In Porto Torres, the environmental analysis of the site starts from the retrieval of information and data collected by the local and national agencies regarding the state of pollution of different environmental compartments over the years.

The collection of data related to air pollution due to industrial emissions has been complex for several reasons: (a) air monitoring, as requested by legislative regulations, primarily targets macro pollutants rather than specific pollutants (i.e., micro pollutants) emitted by industrial activities, (b) data belong to various public and private entities, (c) data are collected for different purposes and on heterogeneous media (47). Data on the presence of contaminants in environmental matrices other than air (soil and water) or on other products (e.g., vegetables grown in the area) are generally scarce. Obtaining information on past air pollution due to industrial emissions is challenging; some sporadic data can be indirectly obtained for poorly water-soluble substances that accumulate in sediments of water bodies.

**Figure 2:**
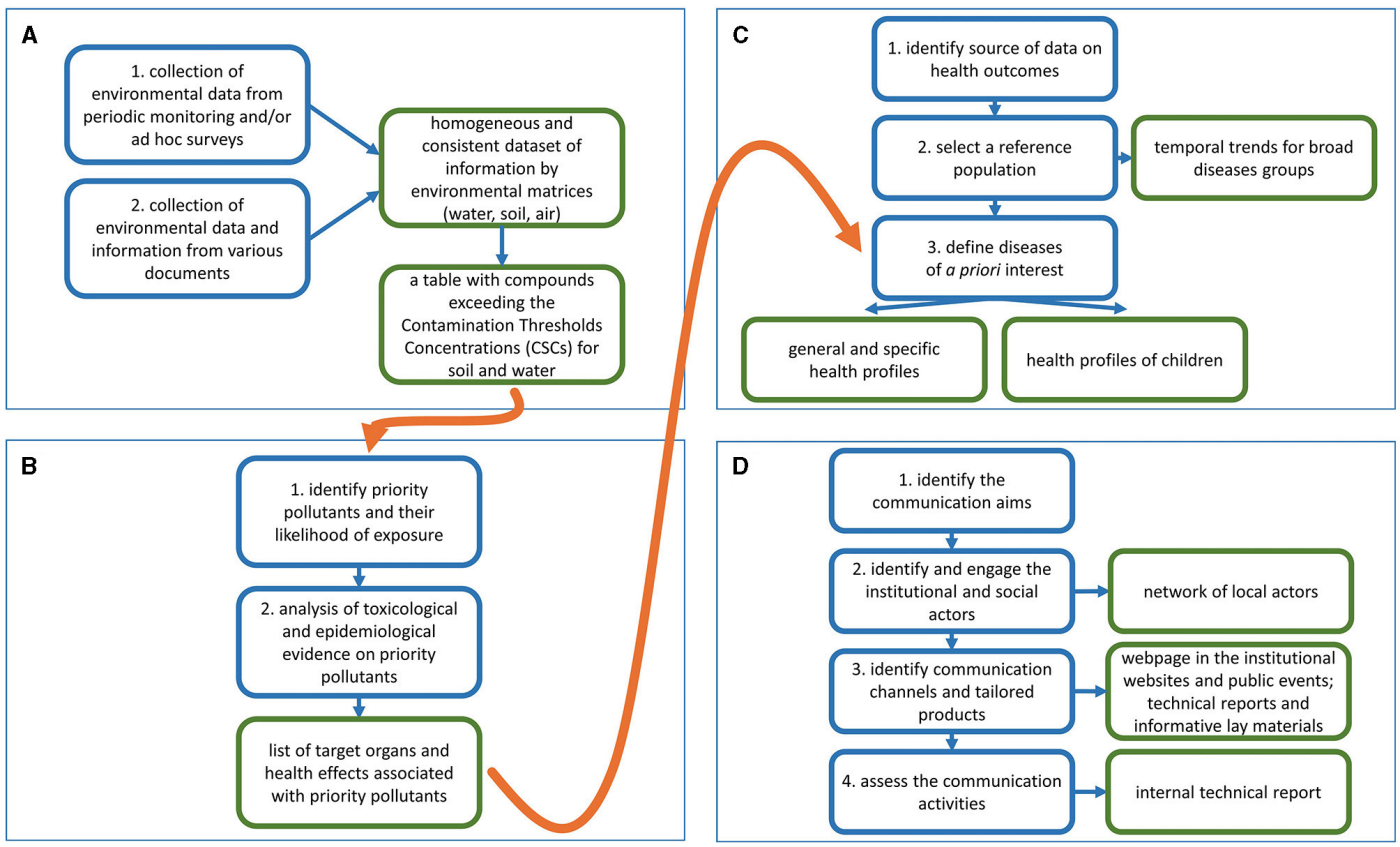
Activities characterizing the domains of the approach (blue border) and their outcomes (green border). **(A)** Collection and systematization of environmental contamination data; **(B)** identification of diseases associated with priority pollutants interest; **(C)** production of health profiles; **(D)** implementation of a participative communication plan.

Figure 2A shows the activities carried out for the collection and systematization of environmental contamination data with their outcomes.

For the air matrix, we start by collecting data measured by the air quality monitoring network, installed according to the criteria set by European regulations, although these data provide a limited understanding of the historical trends of some macro pollutants such as sulfur oxides, nitrogen oxides, total particulate matter, PM_10_ and PM_2.5_, benzene, and some metals, such Pb, Cd, As, Ni. In addition, information on air concentrations of specific pollutants (micro pollutants) stemming from industrial emissions is lacking or limited to temporary and spatially constrained measurement campaigns. In summary, the retrieval of information on air quality, particularly referring to the 1980s and 1990s, was therefore difficult and patchy. Historical estimates of air pollution can sometimes rely on dispersion and fallout modeling allowing identification of the areas of greatest impact, in order to proceed with health and environmental assessment (48). Overall, the analysis of the available documentation provided by the local Authorities evidenced that the absence of an organized monitoring plan, standardized methods and harmonized procedures for data acquisition and storage has led to a heterogeneous data set. Also for soil and water, data were sparse and spotted in time and space. Chemical characterization data should be viewed as “snapshots” capturing contamination levels at the time of sampling, because of these levels may vary over time due to migration phenomena among environmental compartments. Therefore, the systematization and spatial representation of data collected at a specific time may not accurately depict the actual status of contamination over long periods. These data can be considered a compendium of analytical results, useful for providing a qualitative to (semi)quantitative indication of contamination phenomena in the area, evolving in space and time, likely attributable to a massive influx of pollutants into the soil that occurred decades ago (48).

The first outcome of the study is an organic compilation of all the environmental information collected by the various institutions, which allows for the reconstruction of a knowledge framework, albeit a lacunose one, of the various aspects on which local industry has resulted in environmental impact. Closely related to this, we formulate the recommendation for local Authorities to improve/harmonize the monitoring activities, indicating that the sampling data acquisition method would preferably be predefined and computerized.

All the information was systematically included in a single table, listing compounds exceeding the Contamination Threshold Concentrations (CSCs) for both soil and water. This selection was based on qualitative criteria, being able to have only the information about the exceedance of CSC concentrations.

### 3.2 Identification of priority pollutants and potential exposure of the population

To deepen the reading and interpretation of epidemiological data, those contaminants in the area whose presence in the environment may result in exposure for the population (priority pollutants) having specific chemical-physical and toxicological properties of concern for human health were selected. Thereafter, the pathologies of interest, i.e., those associated with exposure to the identified contaminants, were selected based on the available scientific evidence, referring to accredited sources as detailed below and summarized in Figure 2B.

The application of the proposed methodology can represent a step forward to the methodology adopted by the SENTIERI surveillance system. Following this approach, once the priority pollutants have been selected, their toxicological properties, the hazard classification, the dose response characterization, have been gathered from the above cited accredited sources.

Due to the lack of concentration data of contaminants in the environment, as presented in Section 3.1, it was not possible to carry out a quantitative assessment of the exposure of the Porto Torres population, considering different exposure scenarios for the various population groups, as well as aggregated or combined exposure. Only qualitative considerations could be made, highlighting a significant lack of data, which are described in the following.

In Porto Torres, industrial settlements, particularly a large petrochemical plant active for over 40 years, are considered to have caused the main impact on the area. Major pollutants emissions from these plants include aromatic compounds such as phenol, cumene, styrene and chlorinated substances. The area also housed petroleum product depots, cement factories, ironworks with associated asbestos dust emissions, and landfills for the disposal of waste from industrial processing, although no longer in use. The landfills in the industrial area are sources of historical indirect emissions due to the uncontrolled disposal of highly variable types of waste, resulting in soil and groundwater contamination.

Air quality monitoring in the center of Porto Torres has confirmed the presence of several pollutants over the years (i.e., NOx, PM_10_, PM_2.5_, CO, SO_2_). Concentration levels of SO_2_, a maker of industrial emissions, indicate that emissions from the petrochemical plants reached the town center. This conclusion is supported by the analysis of historical meteorological data, which shows that the predominant winds put often downwind the urban area of Porto Torres downwind the industrial emissions.

Population exposure through drinking water, due to the groundwater chemical pollution, was excluded because it is not used for drinking water supply. However, the potential for an oral exposure route has not been completely excluded due to the presence of some –although limited –agricultural areas dedicated to specific crops, possibly affected by deposition of air pollutants. The likelihood of dermal exposure was considered negligible, based on the type of emitted chemicals and the possible exposure scenarios.

Based on the knowledge of the specific industrial cycle and on the qualitative likelihood of exposure, the selected priority substances were: benzene, toluene, xylenes, trichloromethane, vinyl chloride, PCDD/F (polychlorinated dibenzodioxins and polychlorinated dibenzofurans), Polycyclic aromatic hydrocarbons (PAHs), PCBdl, acrylonitrile and heavy metals (arsenic, cadmium, nickel, vanadium, hexavalent chromium, lead).

For each substance, a comprehensive search was carried out to identify the health effects associated with human inhalation and oral exposure. The data were summarized in a table reporting:

- whether the emission is currently active (E);
- the exposure routes (inhalation, I, oral, O);
- exposure likelihood is categorized as either probable (PB), based on available data, or possible (P), where exposure cannot be ruled out. The reasons for defining a possible exposure were: (i) lack of a specific route monitoring data, although the contaminants are known to be present; (ii) presence of known contamination sources. In contaminated restricted zones, the general population was not exposed, but there is a risk of frequent incautious behaviors;
- the target organs/systems on which the contaminants had effects including the related diseases. The differences in health effects resulting from the exposure routes were underlined. The critical effect (generally the most toxicologically relevant effect which occurs at the lowest doses) was highlighted since it is the one used to derive the reference HBV. This allows for considering not only the hazard but also the dose-response relationship. Given the relevance of exposure levels in understanding the plausibility of an association between a pathology and a specific risk factor, the integration of epidemiological and toxicological data was crucial for a sound assessment of the findings.

Table 2 reports such information for benzene, dioxin/furans and arsenic, as examples of the content of the overall table regarding the Porto Torres contaminated site.

**Table 2:** Information for benzene, dioxin/furans, and arsenic on whether the emission is currently active (E), the exposure routes (inhalation, I, or oral, O), the likelihood of exposure (probable, PB, or possible, P) in the Porto Torres contaminated site, and the target organs/systems on which the contaminants have effects including the correlated diseases [modified from Pasetto et al. (23)].

**Pollutant**	**Time**	**Exposure routes**		**Target systems or organs**	**Health effects**
		**I**	**O**		
Benzene	E	P	–	Haematopoietic system	Chronic and low-dose exposure has effects on the blood, with **hematopoietic system** effects considered critical. It causes bone marrow toxicity, leading to a reduction in red and white blood cells, resulting in anemia. Hematological effects have been observed in animals at benzene concentrations in the range of 10–300 ppm (32–960 mg/m^3^ and above), supporting findings in humans (workers) (49). Benzene exposure can also cause bleeding and affect the immune system, increasing the risk of contracting infections. No specific guideline value has been adopted for benzene in air. The International Agency for Research on Cancer (IARC) has classified benzene as carcinogenic to humans (Group 1) (50), and since it is genotoxic, no threshold safe level of exposure can be determined. For general guidance, the concentrations of airborne benzene associated with an excess lifetime risk of leukemia of 10^−4^, 10^−5^, and 10^−6^ are 17, 1.7, and 0.17 μg/m^3^, respectively (51, 52).
Dioxin/ Furans	–	P	PB	Lymphatic system Endocrine system (thyroid) Haematopoietic system	Chronic exposure to low doses can cause damage to both the **immune and endocrine systems (particularly to the thyroid)**. Their interference with the physiological balance of thyroid and steroid hormones, acting as endocrine disruptors, determines effects on fetal development when exposure occurs during pregnancy (prenatal exposure) or in the phases immediately following birth (postnatal exposure) (53, 54). Guidance values have been based on reproductive and developmental effects. In 2002, the Joint Food and Agriculture Organization of the United Nations (FAO)/World Health Organization (WHO) Expert Committee on Food Additives (JECFA) established a provisional tolerable intake of 70 pg/kg body weight per month for PCDDs, PCDFs, and coplanar PCBs applying the Toxic Equivalency Factors (TEFs), based on reproductive endpoints in male offspring of exposed pregnant rats, reflecting a cumulative and chronic rather than acute exposure (55). In 2018, EFSA set a new tolerable weekly intake (TWI) for dioxins and dioxin-like PCBs in food of 2 pg/kg of body weight per week (53), again based on reproductive effects, although the values has been criticized for the questionable robustness of the critical effect (sperm count). According to the International Agency for Research on Cancer (IARC), some dioxins are classified in Group 1 among human carcinogens (56). Some PCDDs and PCDFs can cause tumors of the lymphatic and hematopoietic tissue (responsible for the production of red and white blood cells and platelets), various forms of leukemia, non-Hodgkin lymphomas, and breast cancer. Since these substances induce tumors and likely other effects via a receptor-mediated mechanism, **tolerable intake guidance based on non-cancer endpoints observed at lower doses is considered protective for carcinogenicity** (55, 57). Dioxins and dioxin-like substances are not directly genotoxic. An air quality guideline for PCBs was not established because direct inhalation exposures constitute only a small proportion of the total exposure (1%−2% of the daily intake from food): the main contributor to exposure is oral via the food chain (57, 58).
Arsenic	E	P	P	**Inhalation:** lung, skin, cardiovascular system **Oral :** skin, respiratory and cardiovascular system, bladder	Based on epidemiological studies major effects due to arsenic inhalation include an increase in **lung cancer**, while **skin lesions** and **tumors of the skin, lung, and bladder** are the main and critical adverse effects resulting from long-term ingestion of inorganic arsenic in humans (59, 60). However, years may be necessary for these effects to develop, depending on the level of exposure. Other effects observable at very high doses are linked to developmental toxicity, peripheral vascular and cardiovascular disorders, abnormal glucose metabolism, and diabete (57, 60–64). Furthermore, based on human studies, oral and inhalation exposure to inorganic arsenic levels can cause neurological effects (62). The provisional guideline value in drinking water is 10 μg/L, considering practical difficulties in removing arsenic from drinking water (65, 66). The Joint FAO/WHO Expert Committee on Food Additives (JECFA) determined the lower limit on the benchmark dose for a 0.5% increased incidence of lung cancer (BMDL_0.5_) from epidemiological data to be 3.0 μg/kg body weight per day (2–7 μg/kg body weight per day based on the range of estimated total dietary exposure) (57). As and its inorganic compounds have been classified as human carcinogens by IARC (59).

The critic effects and the relevant information for the risk assessment are reported in bold.

### 3.3 Description of the health profiles and the socioeconomic conditions

The aim of an epidemiological study, as implemented in Porto Torres, is to describe the health profile of the local population as close as possible to the present time. This is done to gather evidence on any possible excess of risk, related to environmental exposures associable with contamination sources' emissions. By identifying such risks, the study aims to facilitate the development of targeted primary prevention measures.

Figure 2C summarizes the activities to provide health profiles and their outcomes as described in the following paragraphs.

The health description has been stratified by sex with a 2-fold aim: (1) to identify risks for sex-related pathologies; (2) to explore disease patterns potentially related to the differential contribution of occupational/residential exposures by sex. Another aspect to be addressed when looking at specific health profiles is the issue of potential variability in susceptibility to environmental exposures among different age groups. To this aim, the study in Porto Torres has been designed to describe the health status of the general population (all ages), as well as of different life stages. Children exhibit distinct activity patterns, behaviors, and physiology that make them differently exposed and/or vulnerable to environmental hazards compared to adults (67–69).

In the Porto Torres case study, the reference population consists of ~305,000 residents from 65 municipalities. The choice of the province as the reference population is justified by several reasons. Firstly, the pool of municipalities of the province belongs to the same administrative unit and geographical context, sharing similar demographic and social characteristics, as well as the same provision of health services. Additionally, mortality and cancer incidence data are provided by the same administrative units, ensuring data reliability in terms of coverage and uniformity of health outcomes coding. Finally, the reference population is large enough (14 times higher than the local community) to guarantee the calculation of robust indicators.

Table 3 exemplifies how to present the specific health profiles. It reports the results of Porto Torres with regard to cancer incidence for diseases identified in the epidemiological literature as being associated, with sufficient or limited evidence, with living close to petrochemical plants. Results shows a number of incident cases significantly higher than expected in males for tumors of the trachea, bronchi and lung, mesotheliomas, and bladder cancer and in females for tumors of the gallbladder and kidney.

**Table 3:** Specific health profile for cancer incidence of the population of Porto Torres considering epidemiological *a priori* evidence (residence and occupation) (2006–2015) [from Pasetto et al. (23)].

**Cancer site**	**Males**			**Females**		
	**OBS**	**OBS-EXP**	**SIR (IC 90%)**	**OBS**	**OBS-EXP**	**SIR (IC 90%)**
Trachea, bronchi, and lung	111	20	122 (104–143)	29	−1	96 (68–130)
Leukemia	21	4	126 (84–181)	13	2	116 (68–184)
Central nervous system	10	0	102 (55-173)	4	−4	52 (18–119)
mesothelioma	7	5	285 (134–536)	< 3^*^		
Skin melanoma	6	−5	56 (24–110)	16	6	154 (97–234)
Gallbladder	11	3	137 (77–227)	15	8	213 (131–328)
Bladder	86	24	139 (115–166)	14	−1	92 (55–143)
Kidney	22	2	108 (73–154)	16	9	218 (137–331)
Multiple myeloma	5	−2	68 (27–144)	4	−5	45 (15–103)

Observed cases (OBS), observed - expected (OBS-EXP), standardized incidence ratio (SIR) estimates x100, 90% Confidence Interval (90% CI).

^*^For privacy SMRs are not computed when the observed cases are < 3.

**Figure 3:**
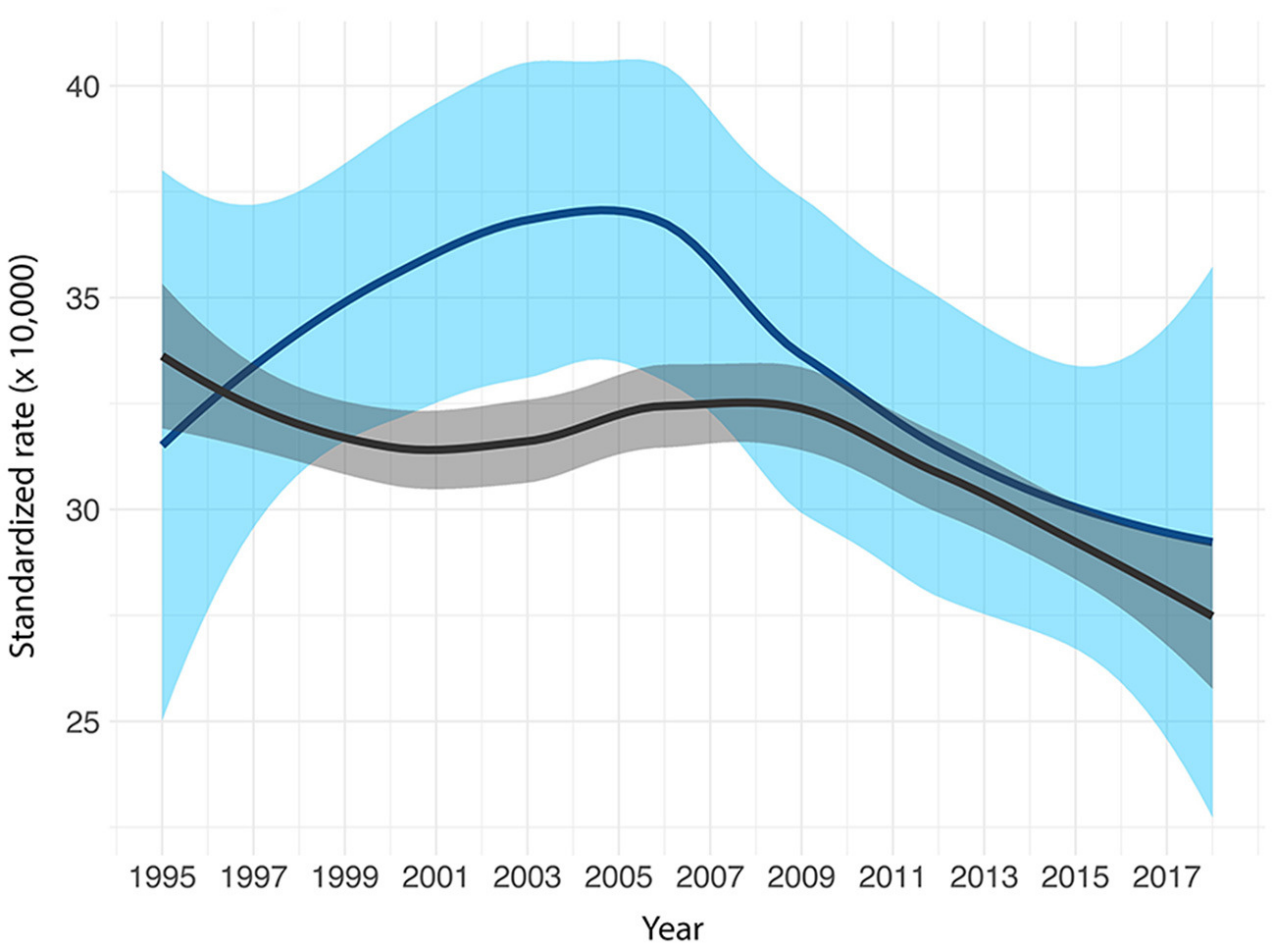
Locally Estimated Scatterplot Smoothing (LOESS) curve and related 90% confidence intervals, Porto Torres (light blue) and reference population (dark gray). Mortality from all cancers, 1995–2018 [from Istituto Superiore di Sanità (70)].

As a further insight, the study on health profiles can be integrated with the analysis of temporal trends for outcomes with the availability of a long time series of data. This kind of analysis allows the identification of changes in disease occurrence over time that may be associated with events or interventions affecting environmental risk factors, such as, for example, emissions from industrial sources, remediation activities, or their health effects. In the case of Porto Torres temporal trends were analyzed for overall mortality and mortality for all tumors by sex. Figure 3 exemplifies the results on temporal trends, reporting figures for males regarding all cancer deaths in the Porto Torres study. It shows a standardized rate in Porto Torres that is significantly higher than in the reference population over the period 1995–2010.

The analysis of local socioeconomic characteristics is an integral part of understanding the health profile and provides valuable contextual insights to understand community's strengths and fragilities (71, 72). The socioeconomic condition represents an important determinant of both exposure to environmental pollution from contaminated sites and related health effects (16). Moreover, in the case of communities residing close to large industrial complexes, socioeconomic evolution is interrelated with the industrial history of the area (8).

Indicators at the community level are of interest and help to understand the local context, together with their interpretation with qualitative information collected through document review and interviews with key informants. This is exemplified by the results regarding the deprivation in Porto Torres, where the community level deprivation indicator showed a very high proportion (around 80%) of the population living in deprived neighborhoods. The applied indicator is a multiple deprivation index representing the following dimensions: unemployment, education level, house ownership and overcrowding. The contribution to deprivation in Porto Torres was almost entirely due to home ownership, with a secondary contribution from unemployment. Analysis of the results with local key informants helped to assess that the impressive proportion of apparent deprivation was largely the result of over-representation of the housing dimension, as a large proportion of the population of Porto Torres lives in public housing. In Porto Torres, this is not necessarily associated with socio-economic deprivation, as public housing was largely promoted in the first phase of industrial development, due to a general lack of housing to accommodate the new workforce. With regard to the other dimensions of deprivation, the relatively high rate of unemployment is due to the loss of jobs as a result of the closure of industrial plants, while the level of education is similar to that of the regional context, as it grew locally during the first period of development associated with the start of industrial activity.

### 3.4 Implementation of a participative communication plan

The aims of the communication plan for Porto Torres were to share the new scientific information and knowledge with the entire community to improve environmental health literacy, and to establish an intersectoral network of local institutional actors to foster participatory communication on environmental health issues. To achieve these goals the following three methodological steps were implemented in terms of the key actions undertaken to realize each methodological step, as summarized in Figure 2D.

#### 3.4.1 Identification and engagement of the local institutional and social actors in the activities aimed at communicating the study findings

Local administrative authorities were identified as the main interlocutors in Porto Torres because of their relevant role in integrating the new evidence from the study into the decision-making process. These authorities were involved from the early stages of the study in *ad hoc* meetings with the study group, in two rounds of semi-structured interviews conducted midway through and after the study and in the co-organization of the final public event of the study to share the findings with the community. The analysis of the information collected in the two rounds of interviews showed a progressive improvement in authorities and community's awareness, particularly regarding concerns about local environment and health issues, conflicts expressed by the different professional sectors, different perceptions of the older and younger community members, and the need to establish a participatory community process (73).

The local health and environmental Authorities were also engaged for selecting the key messages derived from the study findings to be disseminated to the community. In addition, they also proactively participated in the communication process and assumed the responsibility for implementing actions and following recommendations emerging from the study.

Social actors, such as environmental and social organizations and media, are considered relevant stakeholders to be engaged in communication efforts. In Porto Torres, direct relationships were progressively developed with an environmental organization, which played an active role in the final public event. Collaborations between the National Institute of Health and the environmental organization continued after the conclusion of the study to address the issue of environmental justice in Porto Torres, then shared with the community (74).

#### 3.4.2 Selection of communication channels and elaboration of tailored communication materials for diverse stakeholders

In Porto Torres, the selection of appropriate communication channels was a collaborative effort involving the communication experts of regional and local health Authorities. Their institutional websites were selected for broad dissemination of the study findings. All communication materials presenting evidence-based information from the study were accessible through these websites. These materials included a technical-scientific document tailored for environmental, health technicians, and communication experts, lay language materials such as poster information and web texts, a digital map of the study, a glossary of scientific terms used in the study, and a booklet. Online availability of these materials and the direct and transparent communication between researchers and diverse stakeholders, allowed informed participation in collaborative initiatives. The media also benefited from the public availability of evidence-based information for further dissemination of correct and updated information.

#### 3.4.3 Assessment of the impact of communication activities

The assessment of the impact of communication activities requires the identification and the adoption of qualitative and quantitative indicators (75). In Porto Torres the focus was the impact of communication activities on the awareness and preparedness of the local institutional and social actors (73), evaluating improvement in managing information on environmental and health risks. In addition, the tone and content of the information disseminated by the media and the number of articles in regional and local newspapers, websites and social media devoted to the results of the study were considered. The analysis of collected information demonstrated a progressive improvement in community awareness concerning environmental contamination and its associated health effects (73).

**Figure 4:**
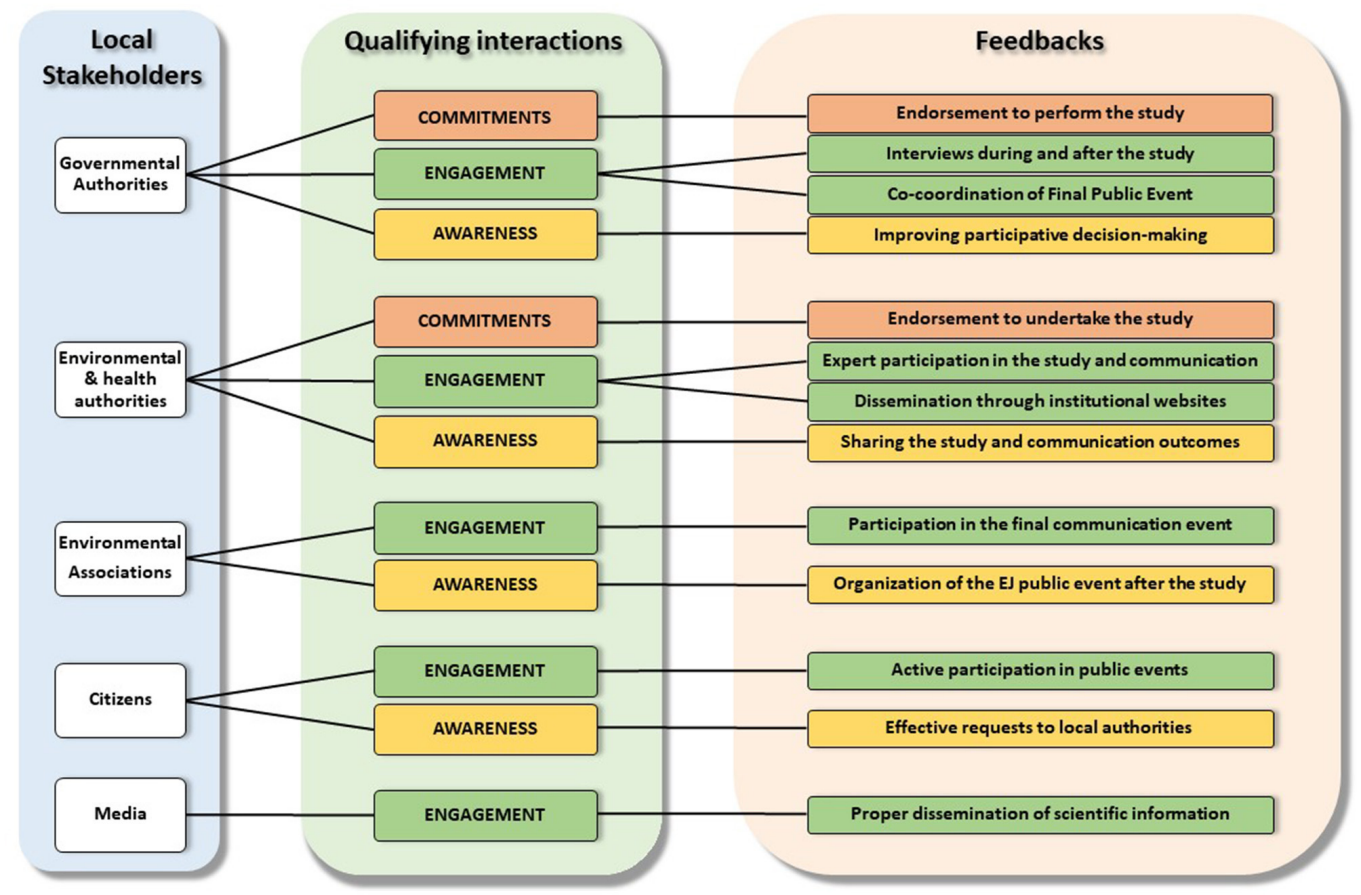
Strategy of engagement with local stakeholders.

The implementation of the communication plan resulted in qualifying interactions for each identified local stakeholder through envisaged strategic activities. Figure 4 illustrates the qualifying interactions with local institutional actors (government authorities, health and environmental authorities) and social actors (environmental associations, citizens, media), as designed in the engagement strategy and adopted during the study activities. It also includes stakeholder's feedback as foreseen in the communication plan, as described in detail elsewhere (73). The implementation of this engagement strategy made it possible to maintain fruitful interactions with both institutional and social actors during the study and after its completion.

### 3.5 Integrated analysis of the study results and their communication to the community

#### 3.5.1 Integrating epidemiological findings with toxicological information

The approach adopted for the Porto Torres study represents a refinement of the SENTIERI surveillance system, overcoming one of its main limitation, i.e., that of neglecting the dose–response curve, thus limiting the analysis to the classification of the chemical substances, which is based only on the hazard (as it is for the IARC classification for carcinogenic compounds, or the classification foreseen by the EU CLP Regulation). The introduction of this further step allows (i) the comparison between environmental exposure levels with established health-based values and (ii) consideration of the possible differences associated with the various sources and routes of exposure, which may be associated with different health outcomes. Indeed, the risk assessment process consists of the identification and characterization (dose-response relationship) of chemical hazards (deriving health-based values below which no significant adverse effects are expected), exposure assessment for the chemicals under evaluation to determine whether these exposures are exceeding the threshold, thus being a concern for public health.

The priority pollutants, as previously explained (Section 3.2), allowed the selection of pathologies of *a priori* interest, in addition to all those selected as typical of the human activity of the area, independently of the local environmental contamination data. Two important aspects must be considered. Firstly, the pathologies analyzed in the specific health profile of the Porto Torres population generally have multiple etiologies and numerous factors may influence their onset, e.g., lifestyle, work activity, socio-economic status and individual susceptibility. Secondly, there is a lack of robust exposure data for the overall population as well as individual exposure data.

For these reasons, it is necessary to interpret the epidemiological results obtained for the pathologies of *a priori* interest reasonably. This interpretation should take into account what is known in terms of exposure to the priority pollutants selected for the studied site and their toxicological profile concerning human health (Table 2).

To better clarify this procedure, two examples are presented for pathologies which, among the causal factors, also recognize exposure to the priority pollutants in the area of Porto Torres.

An excess risk for diabetes mortality was observed in the male gender. Only arsenic is identified as related to diabetes among the selected priority pollutants, primarily following very high occupational exposures. The arsenic hazard characterization indicates that the critical effects at low doses are precancerous skin lesions and renal dysfunctions (Table 2). Given that environmental exposure levels are significantly lower than those in occupational settings, a relationship between arsenic and diabetes should coincide with the presence of the critical health effects (skin lesions and renal dysfunctions) that from a specific analysis of the health profile do not appear to be in excess. This suggests that the observed increase in diabetes can be attributed to other factors, likely non-environmental factors such as unhealthy lifestyles.

For leukaemias, the excess mortality risk in men is accompanied by an excess incidence in both sexes, and in the pediatric age group (four cases observed), with uncertain estimates based on a few cases. However, the presence of excess across various population groups is a notable signal. The priority pollutants that may have contributed based on their hazardous properties are benzene and dioxins. However, the latter could be excluded as exposure to dioxins is primarily associated with the development of thyroid-related diseases (the critical effect at low doses), for which no excess was observed. However, critical effects of benzene affect the hematopoietic system across all exposure levels (Table 2). Data available for the period 2011–2017 on an air sampling station located in the harbor area (near the dock), indicated that the average levels showed contamination peak levels largely exceeding the reference values to protect human health. Although a decreasing trend was noted, data indicated that high levels of benzene persisted in the area for years (76). Hence there is a high plausibility for the association between benzene exposure and leukemia.

#### 3.5.2 Considerations on temporal trends regarding health profiles

The lack of systematic data collection over the years to assess contamination and population exposure does not allow a formal analysis of the evolution patterns of exposure and the potentially related health risk. Nevertheless, the screening of all available information and data on contamination and the assessment of likelihood of exposure of the local population, helps to comment on the results, as it is, for example, for all cancers combined. Figure 3 shows the time trend of all cancer deaths in men in the population of Porto Torres compared with the same trend in the reference population, highlighting a standardized rate significantly higher than in the reference population over the period 1995–2010. The same trend does not occur for women. This result is consistent with a progressive cumulative effect of occupational exposure to several pollutants in the petrochemical industrial complex, where the workforce was exclusively male. The result is also consistent with the excess risks for lung cancer, mesothelioma and bladder cancer, both in terms of mortality and cancer incidence, observed in the study only in men in the population of Porto Torres, considering these diseases as potentially associated with occupational exposure. In addition, the plateau observed in the period 1995–2010 can be explained by taking into account the latency of neoplasms and the operating time of the petrochemical plant [starting in the 1960s, with a progressive reduction in activities (and workforce) from the 1980s onwards], and it is consistent with the results observed in a study of cancer incidence in the cohort of petrochemical workers in the Porto Torres industrial area during the period 1990–2006 (77).

#### 3.5.3 Assessing socioeconomic conditions under the environmental justice lens

The analysis of social determinants of health with community-level indicators helps to assess whether the community is overburdened by factors that may affect its health profile. This is a key step under the environmental justice lens, which requires assessing whether environmental pressures are overrepresented in communities that are disadvantaged within their administrative context (17). The descriptive nature of the study does not allow the differential contribution of environmental and socioeconomic factors to the health profile to be disentangled but helps to understand whether these factors are associated. Furthermore, the reconstruction of the history of the community in association with the development of the industrial complex, through document review and interviews with local key informants, makes it possible to assess whether the community has experienced a significant mono-functional development (i.e., a very closely related socio-economic development of the communities and the local industrial sector), which has excluded or limited other developments. These assessments make it possible to refine recommendations for promoting environmental justice at the local level, considering its socioeconomic and cultural evolution and the related fragilities and resources.

#### 3.5.4 Communication with the target stakeholders

The results of the study should be presented, taking into consideration the targeted stakeholders and the overall communication plan strategy. This may require distinct documents, on one side, aimed at technicians and researchers, and on the other side, for the general population, accessible to both social and institutional actors.

Regarding Porto Torres, two distinct publications were developed in collaboration with local experts engaged in the working group. The two documents were published simultaneously and made available via the websites of the regional and local institutions.

The first document is a technical report elaborated with the regional and local environmental and health technicians, and communication offices. It describes the adopted methodology and the achieved results of the epidemiological study, in its environmental, health, and communication aspects (23).

The second document is a booklet written in lay language, aimed at the Porto Torres community to foster understanding and use of evidence-based information pertaining to the most relevant results and uncertainties of the study (70). This was an action to strengthen the environmental health literacy of the Porto Torres community. The booklet in lay language provides explicit statements on the nature and objectives of the study, including its strengths and limitations. Table 4 summarizes the contents of the booklet with their communication objectives.

**Table 4:** Contents of the booklet in lay language presenting results of the Porto Torres study with the related communication objectives.

**Contents**	**Objectives**
List of institutions and participants in the study activity and collegial meetings by institution	-Clarify the multilevel (national-regional-local) and intersectoral (environment-health-communication) network committed with the community -Specify responsibilities on the study activities and promote accountability of institutions and their personnel
Declaration on conflict of interest with the companies operating or having operated in the industrial complex^*^	Guarantee independence in development and execution of the study activities, including analysis, interpretation and communication of results
Highlights on what the study contributes to and what are its main limitations	Favor discussion among researchers, local institutional and social actors, and citizens on the evidence-based information from the study
Description of the novelties of the study considering the previous available evidence	Point out the relevance of the adoption of an integrated approach including environmental, health, and communication aspects
Description of how the diseases of *a priori* interest characterizing the specific health profile have been selected	Clarify how the study focuses on industrial contamination and health relationships
Description of the study results	Share with the local community the picture on their health profile
Highlights on uncertainties in results	Help in considering uncertainties in reading and commenting on the study results
Main messages regarding environmental health from the study	Focus on those study findings useful to identify priorities for actions
Recommended actions	Foster the local institutional actors to update the integrated approach and for the empowerment of the local community
Commitments of institutions participating in the study with the recommended actions	Provide the community information and references to request actions according to recommendations

^*^No participant declared conflicts of interest.

## 4 Discussion

The outlined approach aims to provide health profiles of communities living close to contaminated sites by integrating different disciplines and methodologies, and by interacting with local institutional and social actors to address local populations' information needs. Commonly, in sites with long lasting industrial contamination, local communities seek answers to questions regarding their health, frequently overlooked. They are keen to understand whether their health is affected by environmental contamination. The proposed approach considers that addressing these questions involves analyzing population health profiles accounting for decades of intertwined evolution between industrial activities and the resulting contamination, along with various other factors characterizing the development of towns and communities, considering health determinants which include socioeconomic factors.

Addressing the health risks and impacts associated with living near industrially contaminated sites is very challenging, especially when the contamination has persisted for decades (3).

### 4.1 Situating the approach within methods applied to assess health risk due to contaminated sites

The main limitations of our approach are inherent to its descriptive nature, and they should be always made clear when presenting the results in similar case-studies. They are: (i) the assessment of population exposure to priority pollutants expressed in qualitative terms; (ii) the possibility that the exposure to some potentially relevant contaminants may not been identified due to gaps in routine monitoring or because pollutants concentrations fall below the limit of quantification of standard methods, a level which may however be relevant for long periods of exposure; (iii) the chance that the combined effects of exposures to multiple contaminants might be overlooked; (iv) the difficulty in retrospectively estimate past exposures to contaminants, especially when the possibly related health effects have a long latency; (v) the multiple etiology of many diseases investigated in the specific health profile.

The above limitations have been highlighted and discussed in reviews of available methods and approaches. Difficulties arise in most cases in assessing the exposure of the population due to long-term contamination processes and in evaluating its health effects (78). Risk assessment and health impact assessment methodologies are available to help estimate the risk and impact of particular contaminants by using defined exposure levels and considering dose-response curves (79, 80). The use of human biomonitoring to estimate exposure to contaminants in industrially contaminated sites have also been discussed, noting that human biomonitoring can only be used to assess exposure to some contaminants and under multiple conditions, and only in some contexts (81). Environmental epidemiology provides the advantage of studying the exposure-response relationships directly in the real-life scenarios, on the other hand, evidence for the causal associations between exposure to contaminants over time and the observed risk at the population level (not from modeling as in risk assessment and health impact assessment) cannot usually be obtained from single epidemiological studies. Savitz suggested that before initiating a new epidemiological study in a contaminated site, it is important to be certain that the expected goals are attainable and that the research itself will support – rather than interfere with – pursuit of needed public health actions (82). He also specified that where data systems are in place, risk assessment combined with epidemiological surveillance – based on routinely collected data - may often be the most efficient, informative response to the exposure event in a contaminated site (82).

Most of the epidemiological studies carried out in contaminated sites use descriptive methods, and analytical epidemiological studies usually focus on contamination of a single environmental matrix by specific contaminants, and do not allow the assessment of the effect of multiple contamination of different environmental media (83). Comba and Pasetto suggested that the “epidemiological characterization” of a given contaminated area, resulting from the application of different study methods, is suitable for assessing causal relationships at the local level and can be seen as analogous to “triangulation” in aetiological epidemiology (i.e., the practice of obtaining more reliable answers to research questions by integrating results from several different approaches, where each approach has different major sources of potential bias that are independent of each other) (84). However, conducting multiple epidemiological studies involves significant economic and time-related constraints, as well as the possibility that waiting for further evidence interfere with the need to manage a threat to public health.

Another aspect to consider is that responding to local people's requests for clarification to improve their knowledge and empowerment in decision-making processes requires an approach that combines the collection of evidence with its effective transfer to and sharing with local stakeholders, by applying participative approaches (85, 86). From a public health viewpoint, the priority is to elucidate complex causal networks for reducing the likelihood of occurrence of environmentally related adverse health effects, with varying degrees of credibility and not to establish causality without any reasonable doubt (87). From this perspective, the above-mentioned limitations in assessing associations between environmental exposure to contaminants and health risk do not undermine the relevance of the proposed approach in highlighting criticalities in the population health profile. Indeed, acknowledging these limitations is crucial for a comprehensive and transparent interpretation of the study results, and for informing future research and environmental public health interventions. The main findings should be considered under the precautionary principle lens, taking into account partial and uncertain evidence when making recommendations to prevent future risks (87).

### 4.2 Strengths and potential improvements of the approach

Notwithstanding the limitations for interpreting causality and in quantifying the local health impact of industrial contaminations, describing the health profiles of resident communities has several advantages. It can be a key step in understanding if these populations are disproportionately affected and whether the overburden can be attributed, at least partially, to environmental contamination. This is assured by addressing different aspects:

- Detailed selection of the diseases of *a priori* interest: the selection of diseases considered in the description of health profiles is based not only on the hazard characterization for each contaminant, but also on assessing the likelihood of exposure in qualitative terms and the routes of exposure (32).
- Comparison with an appropriate reference population: the health profile of the affected community is compared with that of a reference population sharing similar characteristics (same administrative and geographical context, common socio-demographic features, availability of health services, diagnostic and therapeutic care pathways, and lifestyle factors). The detected excess risks are therefore likely to reflect the specificity of environmental pressures and social and socioeconomic specificity of the local communities (24).
- Comprehensive investigation of multiple health outcomes: the approach enables the examination of multiple health outcomes across genders and age classes, with a specific focus on (but not limited to) diseases of *a priori* interest to better explore potential associations with local environmental contaminations (7, 24). Different scenarios, such as excess risks in both sexes or for a specific sex, can indicate different sources of exposure (e.g., outdoor air pollution vs. occupational exposures).
- Consideration of children's health regarded as a potential “sentinel indicator” of environmental risks, especially in industrially contaminated areas, where children may experience high exposures because they usually spend a long time playing outdoors (67, 69). However, as compared to adults, limited epidemiological evidence is usually available for the environmental health effects in children, often because of low statistical power and scarce data, as witnessed by the scant description of environmentally related childhood cancer (67, 69). To this aim, multi-center investigations can provide stronger evidence when large numbers are available.
- Analysis of the observed health profiles considering the known toxicological profile of the priority pollutants: the approach enables the verification of the plausibility of the association between an outcome and the likelihood of exposure, considering the overall toxicological profile including the dose-response curve. This process involves evaluating the consistency between observed health effects and the expected effects based on toxicological data, such as information on the potency, duration, and mechanism of action of the pollutants (31).
- Assessment of socioeconomic conditions and historical evolution of the community: a contextual assessment of the socioeconomic conditions of the local population and nearby industrial activities helps to describe the causes of the health burden (16), although the descriptive nature of the study does not allow disentangling the specific contribution of the often interrelated environmental and socioeconomic determinants.
- Development of an inclusive communication process: a structured communication contributes to fostering environmental public health engaging local stakeholders (52). It promotes environmental justice starting from the recognition of the community's right to be informed through the sharing of the new evidence-based knowledge (27). The adoption of a structured communication through participative activities promotes the responsibility of local institutional actors to discuss with the community the results and recommendations of the study (27, 52).

The adopted approach can be strengthened across all its domains, particularly in reconstructing past environmental exposures. The retrospective assessment of past contamination or emissions/release form point sources often faces uncertainties due to a lack of historical monitoring data, controls, and legally binding procedures. A continuous and widespread air monitoring network in Italy only commenced in the early 2000s and became more systematic after the transposition of the European Air Quality Directive (2008/50/EC) into the Italian Decree no.155/2010. Regulations on industrial permits and emission controls (88) were also introduced around the same time. Consequently, more extensive and up-to-date documentation on industrial plant emissions and controls, as well as air quality monitoring data, is only available for the last 15 years. These data are generally archived by the Ministry of the Environment and Energy Safety or detailed in reports from regional environmental agencies. Additionally, some data may come from company reports, and *ad hoc* environmental surveys and biomonitoring campaigns (e.g., lichen studies). Implementing modeling methodologies to estimate historical contamination and exposures, alongside establishing a systematic and fit-for-purpose monitoring plan, would enhance the understanding of environmental contamination, also considering participative approaches (89).

The informative value of population health profiles can be further improved. Including new health outcomes possibly related to the exposures of interest (e.g., Registry data for congenital anomalies, childbirth certificates, emergency room registries, or estimating some diseases occurrence via prescription drug analysis) alongside a more comprehensive assessment of socioeconomic conditions would provide a richer picture. Socioeconomic data collection could involve citizen questionnaires and deprivation assessments that consider factors like social capital (90).

The Porto Torres study aimed to evaluate community lifestyles using an oversampling approach within the national PASSI surveillance program (91). However, this activity was not completed due to the reallocation of human resources within the Local Health Agency during the COVID-19 pandemic (e.g., contact tracing). Lifestyle evaluation, particularly for vulnerable sub-groups, is crucial for understanding its role in shaping health profiles (92). Additionally, incorporating well-designed, locally-specific questions into existing surveys could improve assessment of environmental pressures, personal habits, and perceptions.

Disentangling the occupational and environmental contributions to community health risk is valuable. Many workers from these communities likely worked in the contaminating industries, potentially experiencing hazardous occupational exposures in addition to environmental risks. Analytical approaches are often necessary (93), although indicators of occupational risk at the community level might sometimes be available or can be provided (94).

Community engagement is another area for improvement. Implementing diverse methodologies and tools, such as participatory workshops on environmental justice, interviews with key informants representing various stakeholders, focus groups with local associations, and the development of interactive tools (e.g., dedicated websites), would foster deeper insight on the community and help citizen engagement (95, 96). Again, limitations due to the COVID-19 pandemic hindered community engagement efforts in Porto Torres.

The final area for improvement concerns the activation of preventive interventions based on health profiles and identified risk factors. This should involve optimizing health policies, both existing and those needing implementation, to address Non-Communicable Diseases (NCDs) and mitigate their effects. Best practices for specific issues, such as secondary cancer prevention through effective screening strategies, should be considered.

All the proposed improvements will be somehow incorporated into a pilot action of the Work Package on Social Inequalities within the European Joint Action Prevent Non-Communicable Diseases, active in the period 2024–2027 (97). This pilot action aims to develop a model for empowering communities residing near industrial contamination to advocate for environmental justice and prevent the health burden associated with cancer and other NCDs. The approach presented here will be continuously updated, ultimately resulting in practitioner guidance informed by new on-site activities and knowledge exchange with researchers experienced in conducting studies across diverse countries and contexts.

### 4.3 Operationalizing the approach and activities to improve equity

In light of the experience conducted in Porto Torres, a descriptive environmental health evaluation of the industrially contaminated-concerned communities should consider the following steps:

a) identifying the main needs/requests from affected local communities through a dialogue with institutional and social actors;

b) selecting environmental, social, and health data from various sources encouraging the use of local information systems to facilitate autonomous development of onsite assessments from local technical bodies. This ensures data reliability in terms of coverage and uniformity of treatment and, in the longer term, provides the basis of a local capacity for integrated environment and health monitoring;

c) integrating different disciplines and methods to define priority pollutants, likelihood of exposure, and risk for diseases of *a priori* interest, with a focus on implementing a trans-disciplinary interpretation of results to produce a comprehensive understanding of the health and socioeconomic profile of the affected communities;

d) empowering local technical stakeholders to build a system that produces health profiles and enables epidemiological monitoring based on the integration of health and environmental data;

e) establishing participative communication plans early and coordinating with local major institutional and social actors;

f) disseminating study outcomes to the scientific community, and communicating with policymakers and all relevant stakeholders on the study findings;

g) identifying interventions, through a systemic and inclusive approach, for environmental, social and health improvements, and addressing key challenges through a priority-based agenda developed with local institutions and social actors;

h) producing environmental public health recommendations for practitioners on research, monitoring and surveillance, interventions and policy actions.

Addressing both opportunities and challenges arising from the involvement of local institutional technical actors in the fields of environment, health and communication, as well as government authorities of communities living in industrially contaminated areas, is a key step. Participatory communication activities contribute to foster inclusiveness and counteract the perpetuation of marginalization mechanisms affecting local communities, thus promoting procedural environmental justice pathways (73, 98). In this regard, environmental health literacy is a central component of local community capacity (44). Indeed, effective access to and the sharing of evidence-based information is the basis for enhancing awareness among local institutional and social actors of the environmental health risks posed by industrial contamination, thus promoting a rebalancing of power in the local decision-making process (99). This empowerment also enhances their capacity to contribute to urban redevelopment (100).

As for directing policy actions to reduce inequalities associated with contaminated sites, these action can be informed through evidence provided by national and local assessments, as suggested by the WHO (101). National monitoring systems, such as SENTIERI in Italy, allow the assessment of environmental health inequalities on a national scale, considering spatial variations and suggesting priorities for action (9).

Additional national interventions can target reducing burdens and inequalities associated with industrial contamination. The mandatory application of Health Impact Assessments (HIAs) holds significant potential. This is particularly relevant as existing industrial areas remain operational, and new polluting facilities may be sited there. In Italy, HIA is mandatory for certain installations, such as large combustion plants, regasification plants, and refineries, as part of the Environmental Impact Assessment procedure (transposition of the European Commission Directive 2014/52/EC in the Italian National Decree No. 104 of 16 June 2017). In the context of contaminated sites, mandatory HIAs prevent further environmental and health burdens, especially in areas and for communities already overburdened by past contamination.

Italy's HIA procedure includes evaluating the community's health profile to assess potential cumulative burdens and their socioeconomic conditions (102). This involves analyzing the pros and cons of the proposed project regarding various environmental justice dimensions at the local level, such as employment, job training, ecosystem resources (land use and water availability), and historical, cultural, and heritage value. Additionally, a cost-benefit analysis is required to assess potential impacts on widening or reducing disparities between subgroups and neighborhoods in terms of environmental health inequalities, including sensory perception (e.g., odors) and psychosomatic conditions.

Priorities can also be established at the regional level, considering not only the health profiles of communities near contaminated areas but also public health resource availability, including healthcare service types, accessibility for communities with the greatest needs, and service quality.

The described approach declines the priorities identified by the WHO for local assessments (96) adding some specificities. Specific features for local monitoring systems were highlighted in the recommendations accompanying the results of the Porto Torres study. Two of them, regarding the local monitoring system, stressed that it should integrate environmental and health data to provide meaningful results, should be open to the public, in the sense that results should be currently available for open consultation, and should be designed to be adaptable to account for modifications to existing industrial plants or new installations. Another recommendation is directed to empowering the local community through initiatives for spreading the results from the study, especially within the schooling context with the involvement of teachers and students. This activity not only allows an increase in awareness of past conditions, but also lays the foundation for community preparedness. Quotes from key representatives of the Porto Torres community, obtained during a focus group held after a local environmental justice event, offer valuable insights (74).

- Environmental justice “rekindles a sense of responsibility in each of us.” Students, in particular, expressed a desire to educate themselves and inform their peers. Environmental justice “gives a broader view of what each person can do in his or her area.” A key question identified by the community is “how to trigger this sense of responsibility.” The community representatives highlighted that environmental issues have often been addressed in a confrontational manner. They believe that framing these issues within the context of environmental justice could facilitate the identification of common goals.
- Looking toward the future, the community representatives emphasized the importance of assessing the feasibility and environmental sustainability of new entrepreneurial activities. They stressed the need for long-term planning, strict adherence to existing regulations, and a high level of attention paid to such activities. Furthermore, they called for “a convergent institutional chain in the objectives for the area,” ensuring clear and coordinated goals.

In essence, the proposed approach provides a framework adaptable for application in other industrial areas facing environmental contamination. This aligns with the objectives of the WHO Collaborating Center for Environmental Health in Contaminated Sites, particularly under the terms of reference *to contribute to WHO's efforts toward assessing the health risks and impacts of contaminated sites and waste, consolidating European collaborative networks, and addressing inequalities and environmental justice*. It merges the needs of Environmental Public Health and Environmental Justice by: suggesting improvements in relationships between researchers, technicians, and community representatives; promoting engagement and empowerment of local institutional and social actors; integrating multidisciplinary knowledge to define recommendations for reducing exposure and health effects; and ensuring accessibility of study results by citizens.

## Data availability statement

The original contributions presented in the study are included in the article/supplementary material, further inquiries can be directed to the corresponding author.

## Author contributions

RP: Conceptualization, Methodology, Writing – original draft, Writing – review & editing. AZ: Methodology, Writing – original draft, Writing – review & editing. DM: Methodology, Writing – original draft, Writing – review & editing. FMB: Methodology, Writing – original draft, Writing – review & editing. II: Writing – original draft, Writing – review & editing, Methodology. MES: Methodology, Writing – original draft, Writing – review & editing. ET: Methodology, Writing – original draft, Writing – review & editing.
